# Comparative Analysis between Three Different Lumbar Decompression Techniques (Microscopic, Tubular, and Endoscopic) in Lumbar Canal and Lateral Recess Stenosis: Preliminary Report

**DOI:** 10.1155/2019/6078469

**Published:** 2019-03-24

**Authors:** Chul-Woo Lee, Kang-Jun Yoon, Sang-Soo Ha

**Affiliations:** Department of Neurosurgery, St. Peter's Hospital, Seoul 135-809, Republic of Korea

## Abstract

**Purpose:**

The purpose of our study is to compare the results of spinal decompression using the full-endoscopic interlaminar technique, tubular retractor, and a conventional microsurgical laminotomy technique and evaluate the advantages and clinical feasibility of minimally invasive spinal (MIS) lumbar decompression technique in the lumbar canal and lateral recess stenosis.

**Methods:**

The authors retrospectively reviewed clinical and radiological data from 270 patients who received microsurgical (group E: 72 patients), tubular (group T: 34 patients), or full-endoscopic decompression surgery (group E: 164 patients) for their lumbar canal and lateral recess stenosis from June 2016 to August 2017. Clinical (VAS, ODI, and Mcnab criteria), radiologic (spinal canal diameter, segmental dynamic angle, and disc height), and surgical outcome parameters (CPK level, Operative time, blood loss, and hospital stay) were evaluated pre- and postoperatively and compared among the three groups by means of statistical analysis. Failed cases and complications were reviewed in all groups.

**Results:**

The mean follow-up period was 6.38 months. The Overall clinical success rate was 89.4%. All groups showed favorable clinical outcome. The clinical and radiologic results were similar in all groups. Regarding surgical outcome, group E showed longer operation time than group M and T (group E: 84.17 minutes/level, group M: 52.22 minutes/level, and group T: 66.12 minutes/level) (p<0.05). However, groups E and T showed minimal surgical invasiveness compared with group M. Groups E and T showed less immediate postoperative back pain (VAS) (group E: 3.13, group M: 4.28, group T: 3.54) (p<0.05), less increase of serum CPK enzyme (group E: 66.38 IU/L, group M: 120 IU/L, and group T: 137.5 IU/L) (p<0.05), and shorter hospital stay (group E: 2.12 days, group M: 4.85 days, and group T: 2.83 days) (p<0.05). The rates of complications and revisions were not significantly different among the three groups.

**Conclusions:**

MIS decompression technique is clinically feasible and safe to treat the lumbar canal and lateral recess stenosis, and it has many surgical advantages such as less muscle trauma, minimal postoperative back pain, and fast recovery of the patient compared to traditional open microscopic technique.

## 1. Introduction

Traditional treatment of spinal stenosis has been wide laminectomy involving undercutting of the medial facet and foraminotomy [1]. With the introduction of the operating microscope, laminectomy was refined, widely accepted by spine surgeons. More limited decompressive procedures including bilateral foraminotomies and unilateral approaches to bilateral decompression have been shown to be effective [2–4]. Nonetheless, tissue-sparing procedures are becoming more common. Among those MIS decompression techniques for the lumbar canal and lateral recess stenosis, techniques by using tubular retractor and percutaneous endoscope have been reported to have many surgical advantages such as less postoperative back pain and benefits for rehabilitation [3, 5–12]. However, its clinical efficacy and safety were not proved to the extent to satisfy most spine surgeons. The present study was undertaken to retrospectively compare the results of spinal decompression using the full-endoscopic interlaminar technique (group E), tubular retractor (group T), and a conventional microsurgical laminotomy technique (group M) with a goal to evaluate the advantages and clinical feasibility of MIS lumbar decompression technique in lumbar central spinal stenosis. To the best of our knowledge, this is the first comparative study to analyze the three methods (endoscopic, tubular, and microscopic) and give the answer to the question what the advantages of MIS decompression technique compared to previous open laminectomy are in the treatment of lumbar canal and lateral recess stenosis.

## 2. Materials and Methods

This study was approved by the relevant institutional review board.

### 2.1. Patient Population

277 patients were enrolled by inclusion and exclusion criteria. Seven patients (group E: five patients, group M: two patients) were dropped out due to several reasons (reluctant to further visit to out patient clinic, cannot touch by call). A retrospective review was performed on 270 patients (187f, 83m) who had undergone full-endoscopic (164 patients), tubular (34 patients), and microscopic (72 patients) laminotomy and flavectomy, for degenerative lumbar central or lateral recess stenosis between June 2016 and August 2017 at a single center. Inclusion criteria were patients who were preoperatively diagnosed with lumbar central canal or lateral recess stenosis with the symptoms of neurogenic intermittent claudication (NIC) and radiculopathy and refractory to conservative treatment at least for three months. Segmental instability, degenerative spondylolisthesis more than Meyerding Grade I, multidirectional rotation slide, and Scoliosis more than 20 degrees, combined foraminal stenosis in the same or lower level or coexisting pathologic conditions such as acute inflammation, infection, or tumor, were excluded. There were no significant differences in preoperative data between different three groups except the total number of patients according to the technique. Patient's demographics and characteristics are summarized (Table 1).

### 2.2. The Methods of the Technique Selection

Three spine surgeons (CW Lee, SS Ha, KJ Yoon) in single center performed the surgeries which were recruited in this study. Each of three surgeons selected the single decompressive method which was the best at their own hands for all their patients (CW Lee: endoscopic surgery, SS Ha: Tubular surgery, and KJ Yoon: microscopic surgery). All three surgeons who performed surgeries in this study had already a great deal of traditional spinal surgery experience (over 5000 cases). But, there was some difference in the number of each of the cases which each surgeon had experienced before the study (CW Lee: 42 endoscopic lumbar decompressive surgery cases, SS Ha: 612 tubular lumbar decompressive surgery cases, and KJ Yoon: 1235 microscopic lumbar decompressive surgery cases).

### 2.3. Surgical Technique

General operative descriptions are given below for each type of procedure. All the patients underwent general or epidural anesthesia with sedation. The patients were placed in a prone position with positioning pads under the shoulders and superior iliac crests. The affected level was verified by intraoperative C-arm fluoroscopy. The operation was performed bilaterally via a unilateral access using an “undercutting technique.”

#### 2.3.1. Endoscopic Decompression

Percutaneous endoscopic laminotomy with flavectomy by uniportal, unilateral Approach for the lumbar canal or lateral recess stenosis was previously introduced by authors [13]. Endoscopic: The operative procedure was performed by using a complete endoscopic instrument system: Ilessys Delta® (joimax GmbH, Raumfabrik 33A, Amalienbadstraße, 76227 Karlsruhe, Germany) or Vertebris stenosis (Richard Wolf GmbH, Knittlingen, Germany). After a paramedian skin incision approximately 9 mm long which targets the caudal margin of the upper lamina, blunt insertion of a serial dilator was followed. The operation sheath over the dilatator was inserted with the beveled opening directed medially toward the ligamentum flavum. Under the endoscopic direct visual control, ipsilateral decompression was performed first by means of craniocaudal laminotomy and partial facetectomy with endoscopic drills and punches. The contralateral side was entered dorsally to the dura. The ligamentum flavum was initially left intact as a protective barrier for the dura and neural components. Contralateral bony structure including partial lamina and facet was decompressed. Subsequently, ligamentum flavum was removed in en bloc fashion. The decompression was finished when the decompressed dura and spinal nerves were clearly seen on both sides. On a case by case basis, disc herniation to compress the neural structures was removed. The incision was sutured in a subcutaneous layer with Vicryl followed by Dermabond on the skin edge.

#### 2.3.2. Tubular Decompression

The tubular decompression for spinal canal and bilateral lateral recess stenosis with unilateral approach is described in detail elsewhere [14]. An 18 mm paramedian horizontal skin incision was then made. The muscle was sequentially dilated, after which we placed an 18 mm working channel of the shortest length that would allow the adequate depth of access (usually 50 or 60 mm). The operative microscope was moved into the field, and the laminar edge was identified. A laminotomy was performed, extending cephalad above the insertion of the ligamentum flavum on the inferior surface of the superior lamina (to ensure adequate resection of ligamentous compressive elements) and caudally to include a smaller portion of the superior aspect of the inferior lamina exposing the pedicle. Resection of the medial facet complex was performed as it is necessary to decompress the lateral recess and the foramina adequately. The working channel was then angled medially to expose the anterior aspect of the spinous process, which was then removed utilizing a drill. This procedure exposed the lateral recess on the contralateral side where the residual lamina and ligamentum flavum could be resected using the drill, Kerrison punches, and curettes. The angle of approach is the same as that commonly taken during an open laminectomy that allows undermining of the contralateral facets, making the anatomy familiar to most spine surgeons. Satisfactory decompression of the lateral recess and foramina is achieved under direct visualization. The incision was closed in layers with Vicryl followed by Steristrips.

#### 2.3.3. Microscopic Decompression

The microsurgical procedure of unilateral approach with bilateral decompression (ULBD) is described in detail elsewhere [6, 15]. Surgery was performed in a standardized manner with a minimally invasive approach via a unilateral laminotomy with partial resection of the inferior aspect of the cranial hemilamina and, usually to a minimal degree, from the superior aspect of the caudal hemilamina. After the ipsilateral decompression, the base of the spinous process was undercut by medial angulation of the operative microscope, and the contralateral hemilamina together with the hypertrophied medial facet was partially removed after bilateral flavectomy, and the lateral recess and neural foramina were decompressed contralaterally. Care was taken not to detach the spinous process completely and to preserve the hypertrophied ligamentum flavum as long as possible for the protection of the dural sac and nerve root during drilling.

The difference of 3 decompression techniques was summarized and compared (Table 2).

### 2.4. Radiographic Analysis

**Figure 1:**
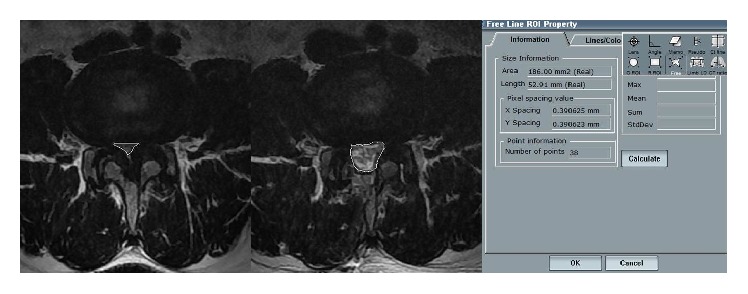
MRIs showing pre- and postoperative change of dural sac cross sectional area using an automated and digitalized tool in the PACS system.

Radiologic measurements were done using automated and digitalized tools in the PACS system, PiView 1.0 (Infinitt Co. Ltd., Seoul, Korea). To evaluate the degree of decompression radiologically, the cross-sectional area of dural sac at the disc level was measured for the preoperative and postoperative MRI, by using the digitalized tool (Figure 1). Spinal canal dimension was investigated and compared pre- and postoperatively by axial MRI image at middisc level. All patients underwent functional X-rays both preoperatively and at the end of the follow-up period.

### 2.5. Outcome Measures

Patients were evaluated pre- and postoperatively with the Visual Analog Scale for leg and pain, Oswestry Disability Index scores, and the modified MacNab criteria. Postoperative patient satisfaction survey, which was composed of two questions, was also performed. Serum creatine phosphokinase (CPK) enzyme was measured before the operation and a day after the operation to investigate the degree of iatrogenic muscle injury according to the operative methods. Complications related to the surgery and surgical outcomes such as operative time, hospital stay, and blood loss including postoperative hemovac drainage, were reviewed.

### 2.6. Statistical Analysis

Statistical analysis was conducted using SPSS. All intra- and intergroup comparisons were conducted using a student's t-test, paired t-test, one-way ANOVA, and chi-squared test as appropriate. Statistical significance was accepted at p<0.05.The materials and methods section should contain sufficient detail so that all procedures can be repeated. It may be divided into headed subsections if several methods are described.

## 3. Results

### 3.1. Clinical and Functional Outcomes

**Figure 2:**
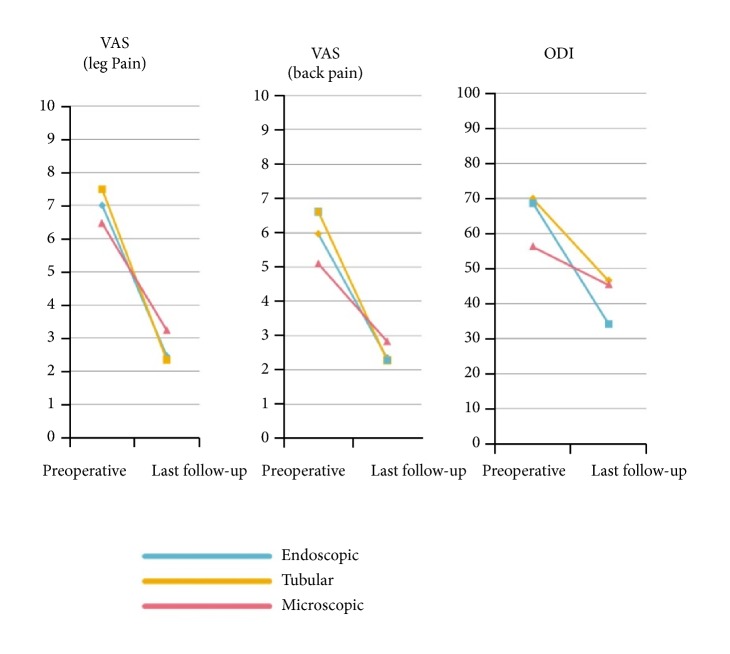
Pre- and postoperative change of VAS and ODI.

The average follow-up duration was 6.38±4.35 months. The three groups had comparable VAS and ODI scores preoperatively. At the last postoperative follow-up, similar statistically significant improvements in VAS and ODI outcome scores were found (VAS (back pain-) group E: 5.97-2.35, group T: 6.61-2.28, group M: 5.09-2.83; VAS (leg pain-) group E: 7.01-2.46, group T: 7.48-2.33, group M: 6.47-3.24; ODI-group E: 69.8-46.5, group T: 68.6-34.2, and group M: 56.3-45.3) (Figure 2).

**Figure 3:**
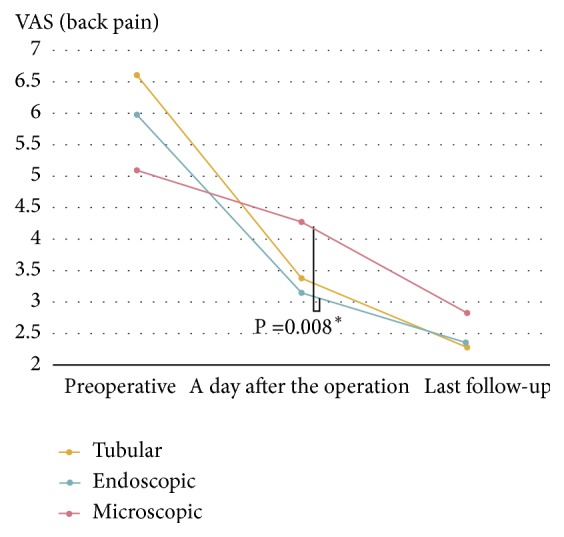
The difference of immediate postoperative back pain. VAS=Visual Analogue Scale and F/U= Follow-up.

All three groups showed favorable postoperative clinical outcomes. However, VAS score for early postoperative back pain which was evaluated at a day after the operation showed less postoperative back pain in groups E and T compared with group M (group E:3.13, group T: 3.38, and group M: 4.28). The difference between group E and M were statistically significant (p=0.008) (Figure 3).

**Figure 4:**
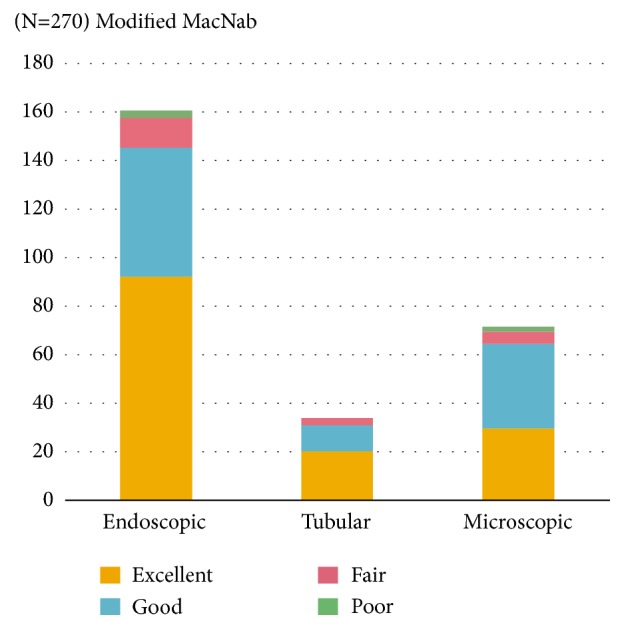
Clinical outcome by modified McNab criteria.

At the final follow-up review, the modified MacNab criteria were rated as follows: excellent in 142 patients (71.3%) (group E: 92 patients, group T: 20 patients, and group M: 30 patients), good in 99 patients (22.5%) (group E: 53 patients; group T: 11 patients, and group M: 30 patients), fair in 21 patients (4.2%) (group E: 13 patients, group T: 3 patients, and group M: 5 patients), and poor in 5 patients (1.9%) (group E: 3 patients, and group M: 2 patients). Therefore, 93.8% of the all patients answered excellent or good results. Overall success rate was similar among the three groups (group E: 88.4%, group T: 91.1%, and group M: 90.2%) (Figure 4).

**Figure 5:**
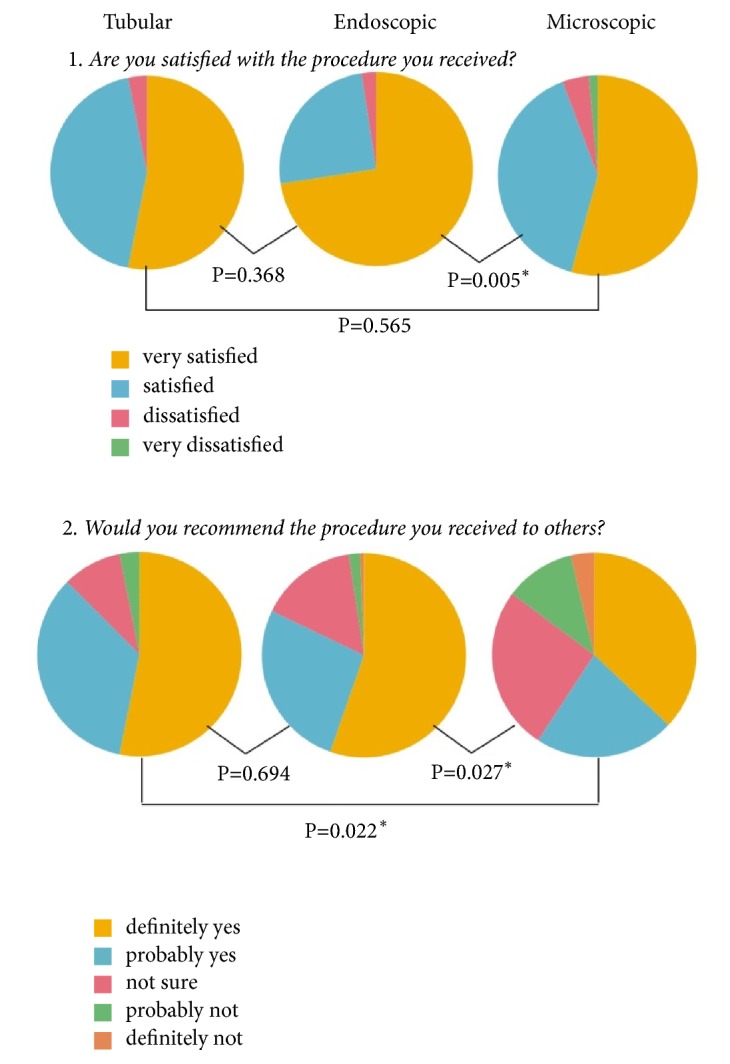
Patient satisfaction.

From the patient satisfaction survey, 257 patients (95.1%) (group E: 160 patients (97.5%), group T: 31 patients (91.1%), and group M: 66 patients (91.6%)) reported subjective satisfaction and 208 patients (77.0%) (group E: 138 patients (84.1%), group T: 28 patients (82.3%), and group M: 42 patients (59.7%) ) responded that they would recommend this procedure to others. The patients in groups E and T gave more positive responses on the satisfaction survey than group M and the differences in patient responses between groups E and M to both questions and between groups T and M to the second question were statistically significant (Figure 5).

### 3.2. Radiological Results

Dural sac expansion was observed by the comparison of pre- and postoperative MRI axial images. It was statistically significant in all groups. However, our study showed that there was no significant difference among the three groups in the amount of decompression (Table 3). There was no case of postoperative increased kyphosis, instability, and decreased disc height in the operated segment.

### 3.3. Surgical Outcomes and Perioperative Complications

Even though the differences were statistically insignificant, MIS decompression group (groups E and T) showed less blood loss (group E: 35.4 ml, group T: 72 ml, and group M: 134.3 ml). Patients in group E experienced average shorter hospital stays and longer operation times than those in groups T and M (Hospital stay-group E: 2.12 days, group T: 2.83 days, group M: 4.85 days, and p≤0.001; operation time-group E: 84.17 minutes/level, group T: 66.12 minutes/level, group M: 52.22 minutes/level, and p≤0.001).

**Figure 6:**
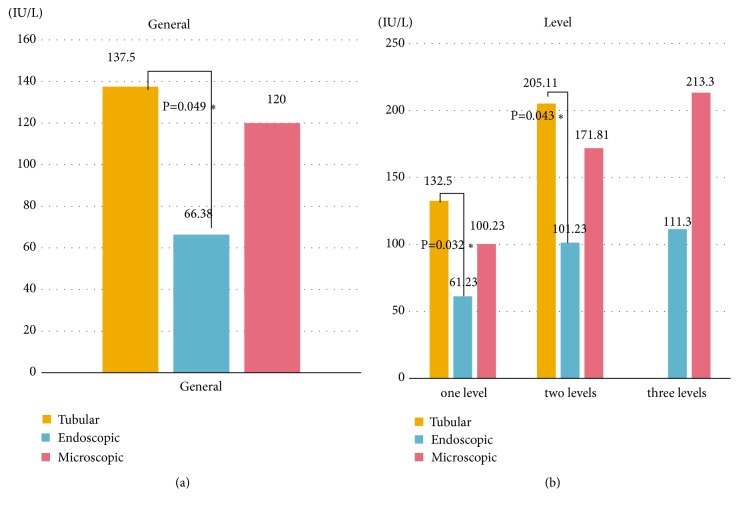
Postoperative increased amount of serum creatine phosphokinase (CPK): (a) comparison between 3 different decompressive methods; (b) comparison between 3 different decompressive methods according to the number of decompressed level.

The results for postoperative changes of serum creatine phosphokinase (CPK) showed that, in general, tubular decompression group had significant increase of serum CPK enzyme compared to endoscopic decompression group (group E: 66.38 IU/L, group T: 137.5 IU/L, group M: 120 IU/L, and p=0.049). Also, endoscopic decompression group showed less increase of CPK enzyme compared to microscopic decompression group, although it was statistically not significant; as the number of decompressed levels increases, such an inclination was more evident (one level-group E: 61.23 IU/L, group T: 132.5 IU/L, and group M: 100.23 IU/L; two levels-group E: 101.23 IU/L, group T: 205.11 IU/L, and group M: 171.81 IU/L; three levels-group E: 111.3 IU/L, and group M: 213.3 IU/L) (Figure 6).

The results showed that there was no significant difference in morbidity rates associated with the procedures (group E, 7.9%; group T, 8.8%; group M, 8.3%). Total of 12 patients (group E: seven patients, group T: one patient, and group M: four patients) suffered from postoperative transient dysthesia in the same preoperative dermatomal distribution. Those patients were given selective nerve block and oral gabapentin medication. Their symptoms improved over a 3-month period. There was one case of motor weakness in endoscopic decompression but it recovered to normal status three months later. Five cases of dura tear were reported (group E: four cases, group M: one case). One case of dura tear, which occurred during the microscopic decompressive procedure, was repaired by revision surgery. Four other cases of dura tear from endoscopic decompressive surgery did not cause further negative consequences and needed secondary repair surgery. Among total cases, there were 7 combined discectomy cases (E group: 3 cases, T group: 2 cases, and M group: 2 cases). Relative high percentage of discectomy cases in tubular decompression group was seen (E: 1.8%, T: 5.8%, and M: 2.7%). It was statistically not significant due to small number of cases. The same revision surgery methods treated two cases of disc reherniation from endoscopic and tubular decompressive surgery. No patient had the revision surgery for the incomplete decompression (Table 4).

## 4. Discussion

Various therapeutic modalities ranging from open laminectomy to minimally invasive decompression were introduced as the surgical treatments of lumbar canal and lateral recess stenosis. Several decompressive techniques have been developed following the MIS concept to minimize iatrogenic injury and preserve segmental stability. Many studies have reported more favorable clinical results with MIS decompressive techniques than traditional methods [3, 5, 6, 16–18]. Today, percutaneous endoscopic spinal surgery has become a standard treatment in various lumbar spinal diseases ranging from a simple contained disc to complicated cases such as highly migrated disc herniation. The spinal stenosis in the canal and foramen can now be operated fully with endoscope [9–11, 19–23]. However, previous studies have also presented that MIS techniques have their own limitations such as stiff learning curves and relatively high complication rates, compared to conventional techniques [5, 12, 24–31]. Some authors have reported successful clinical results of MIS decompressive techniques with the tubular system and full endoscopic system for lumbar stenotic disease [9–11, 29, 32], but apart from these limited studies, there are few reports that explain or convince most spine surgeons of the effectiveness and clinical feasibility of MIS decompressive techniques for the lumbar canal and lateral recess stenosis.

The purpose of this study was to compare the results of spinal decompression using the full-endoscopic interlaminar technique (group E), a tubular retractor (group T) and a conventional microsurgical laminotomy technique (group M), and evaluate the advantages and clinical feasibility of MIS lumbar decompression technique in lumbar central spinal stenosis.

### 4.1. Clinical Outcome and Patient's Satisfaction

Several clinical parameters such as VAS and ODI showed significant clinical improvement postoperatively in all groups. These clinical results were similar among the three groups and comparable to those obtained from previously described microsurgical or tubular techniques and corresponded to data reported in the literature [2, 5, 6, 33].

Interestingly, although such clinical parameters showed similar results among the groups at final follow-up, the immediate postoperative results showed that MIS decompression groups induced significantly less back pain compared to the traditional microscopic decompression technique. All patients were treated by same analgesic protocol after the operation regardless of type of the decompressive technique. Only NSAIDs (ibuprofen 400mg, PO, and Bid) were given to the patients 6 hours after the operation until discharge We added other pain killer (piroxicam 20mg, IM, and PRN) if patients complain unbearable postoperative pain during admission (group E: one patient, group T: one patient, and group M: three patients ). But, most patients did not need additional analgesics. We assert that these findings reflect the less tissue damage and minimally invasive nature of the MIS decompression technique, and such a less immediate postoperative back pain was one of the merits of MIS decompression technique.

In this study, another identified advantage of MIS lumbar decompression was the high level of satisfaction by the patients. MIS decompression groups had more positive responses to the satisfaction survey than the microscopic decompression group, and such results in MIS groups exceed those reported for previous other lumbar decompression techniques [4, 5, 7, 34]. These findings appeared to be related not only to the minimal operative skin scar from MIS technique but also to the minimal immediate postoperative back pain, short hospital stays from fast recovery, and early return to normal life owing to the minimal invasiveness of MIS decompression, which are all mentioned in previous articles as merits of MIS surgery [7, 25, 32, 35, 36].

### 4.2. Decompression Ability and Radiological Outcome

Previous reports have presented that one of the drawbacks in MIS bilateral decompression via unilateral approach is incomplete decompression, especially, contralateral root decompression [37, 38]. It is due to very limited operative view and working space to manipulate the surgical instruments during the operation. However, the results of this study showed competent decompression ability of MIS techniques equal to the traditional microscopic technique. In the current study, the radiologic analysis of the canal diameter changes proved the satisfactory decompression ability of MIS techniques. There was no revision case in groups E and T due to the incomplete decompression, which also supports the efficacy of MIS decompression in the lumbar stenosis. In the percutaneous endoscopic lumbar decompression, the limited surgical visibility through the endoscopic channel and the unfamiliarity with the use of endoscopic instruments can prevent complete decompression of spinal canal and bilateral recess area during the early stage of the learning curve.

Intraoperative bleeding, although it is minimal, can induce blurred operative view, which also could be the obstacle to proceed with the decompressive procedure. However, as we became familiar with the endoscopic lumbar anatomy and the basic usage of the endoscopic instruments such as high speed drills and punches, we were able to perform a thorough bilateral decompression. Strict bleeding control by RF bipolar and proper adjustment of hydrostatic pressure by irrigative pump system were the keys to maintain a clear operative view until complete decompression was achieved. Variable endoscopic operative views caused by tilting and rotating the endoscope enabled complete exploration around the main pathology without difficulty.

### 4.3. Serum CPK

The role of elevated serum CPK levels as a biochemical indicator of muscle injury has been shown in previous studies. A significant reduction in postoperative creatine phosphokinase was reported among participants treated with MIS techniques when compared with conventional laminectomy [39–41]. In this study, endoscopic decompression group showed the tendency of less increase of CPK enzyme compared to microscopic decompression group. Although it was statistically not significant, considering such an inclination was more evident as the number of decompressed levels increased, we assert that endoscopic decompression technique has more advantages to save paraspinal muscle damage than traditional microscopic decompression technique. Further study by recruiting more patients to this data would be needed to prove significant less invasiveness of endoscopic decompression technique compared with traditional laminectomy by the parameter of serum CPK.

Previous several authors reported the variable patterns of serum CPK change in tubular lumbar decompression, but the relationship between increased serum CPK level and postoperative lumbar back pain remained controversial [6, 16, 32]. Curiously enough, in this study, tubular decompression group showed significantly more increase of serum CPK compared to endoscopic decompression group. We think it may be related to the difficulty in inserting the working tube in the minimally invasive way or be caused by the initial surgical step to remove some parts of the muscle inside the tube after the insertion of a tubular retractor to acquire clear operative field. Although such change of CPK in tubular decompression group did not affect the postoperative clinical outcome, such as immediate postoperative back pain and hospital stay, compared to other two groups in this study, this finding is worthy of the attention.

### 4.4. Learning Curve and Operative Time

Most MIS techniques have steep learning curves and need longer operation time, especially, in the early stage of the learning curve [5, 28, 42, 43]. MIS techniques of ULBD have a very narrow vision and physical space inside the cannula which has a small diameter. Such limitations can cause prolonged operation time and intraoperative complications. Particularly with the endoscopic lumbar spine surgery, beginner surgeons who are not familiar with endoscopic surgical anatomy have difficulty manipulating endoscopic operative equipment, which can lead to long operation time. In this study, mean operative time in endoscopic decompression group was longer (E: 84.17 minutes/level, T: 66.12 minutes/level, and M: 52.22 minutes/level) than those in the other two decompression techniques. This was due to the surgeon who performed the endoscopic decompression was in the learning curve. However, the chronological analysis of the operative time in the endoscopic decompression group showed the operative time decreased with more cases (initial third (55 cases): 102.1 minutes, second third (55 cases): 85.9 minutes, and last third (54 cases): 66.60 minutes). Percutaneous endoscopic lumbar decompression is a complex and technically demanding procedure associated with a steep learning curve and needs considerable experience to achieve an adequate neural decompression. However, from reviewing the chronological change of the operative time in the endoscopic decompression group, we could conclude that the endoscopic decompression technique has reasonable operative time compared to other two techniques and can be learned with time.

### 4.5. Perioperative Complications

There have been concerns about a number of potential disadvantages and complications in the MIS decompression techniques for the lumbar stenosis. Some authors have asserted that limited visualization of the critical neural structures and the difficult handling of operative instruments in MIS decompression techniques may be responsible for the higher rates of complications such as dura tear or neural injury. However, in this study, the incidence of surgery-related complications in MIS decompression groups was not high compared with the microscopic decompression group (group E: 7.9%, group T: 8.8%, and group M: 8.3%) and comparable to those reported in previous studies of other MIS decompression techniques [3, 6, 9–11, 15, 30, 44]. Considering the surgeon who performed the endoscopic decompression was in the learning curve, these results reflect MIS surgery to be a relatively safe and reliable method to decompress stenotic spinal canal and lateral recess. In the current analysis, there were six cases of dura tears (2.4 %) in group E, no case in group T and a case in group M (1.3%). One case of dura tear in microscopic decompression group was managed with suture with No 5. Prolen. Most cases of dural tears in endoscopic decompression were repaired by applying a gelfoam and TachoSil sealant patch (Baxter Healthcare Corporation, Deerfield, IL, USA) during the operation and ABR afterwards, because those were small nicks. Although the incidence of dura tear in endoscopic decompression group was higher than other groups, most cases occurred in the surgeon's early stage of learning curve, and its incidence was comparable with previous literature findings [5, 9, 10, 17, 30, 44, 45]. Constant saline irrigation through a working channel provided more epidural working space between the neural structures and the surrounding soft tissues during the endoscopic decompression, which made it easy to differentiate and manipulate the related structures in the narrow operative fields. Such an advantage of the acquisition of better intraoperative view by irrigative pressure in the endoscopic decompression helped to decrease the complication rate. No case in the endoscopic decompression group resulted in negative consequences such as persistent CSF leakage or revision surgery in this study.

We identified a total of 12 cases of transient postoperative dysthesia and a case of motor weakness in this study. There was no significant difference in the incidence of neural injury among the three decompression techniques. Reviewing these cases, excessive retraction of the neural structures without adequate adhesiolysis was considered as the major cause of the neural injury regardless of the decompressive methods. The usage of RF bipolar with high intensity was another cause of postoperative leg discomfort in the endoscopic decompression group. Minimal and delicate manipulation with beforehand adhesiolysis of the neural structures is important in achieving a favorable clinical outcome without intraoperative neural complications. Careful RF bipolar coagulation with adequate intensity is recommended to avoid postoperative dysthesia in the endoscopic decompression.

### 4.6. Limitations

This study was a retrospective study and not a randomized one with the different size of samples among the three groups. A prospective randomized study that compares each procedure with standardized preoperative data, which have even numbers of cases among groups, is required. Despite such shortcomings, current study showed obvious results that MIS decompression techniques have comparable outcome with traditional microscopic decompression technique or even superior outcome such as less immediate postoperative back pain and high patient satisfaction and acceptable complication rate compared with those of previous studies. Another weakness of this study is that the follow-up period was rather short. The real advantages of MIS techniques should be proven not only by short-term clinical and radiological outcomes (less immediate back pain, less increased CPK enzyme, and sufficient spinal canal decompression) but also longer-term results which can give real benefits to patients. The issues, compared to the traditional decompressive surgery, whether the minimally invasive decompressive surgery is advantageous to decrease the incidence of secondary operation due to postoperative instability and postoperative chronic back pain or not should be addressed in future long-term studies with more patients.

Each procedure was performed by three different surgeons. It may be attributed to intersurgeon variability in terms of experience and case load. Ideally, all cases should be performed by the same surgeon to minimize the influence of personal experience. However, all surgeons who performed surgeries in this study had already a great deal of traditional spinal surgery experience. Although the surgeon who performed the endoscopic decompression was in his learning curve, it was encouraging to find that the results from the endoscopic decompression group were comparable with the two other groups, which can imply good clinical feasibility of MIS technique. Although this study has many limitations, thinking collectively from the overall results, authors think that the results of this study suffice to prove the efficacy and clinical feasibility of MIS decompression techniques in the lumbar canal and lateral recess stenosis and to convince spine surgeons to apply this technique in their practice.

## 5. Conclusions

MIS lumbar decompression technique is clinically feasible and safe to treat the lumbar canal and lateral recess stenosis, and it has many surgical advantages such as less muscle trauma, minimal postoperative back pain, fast recovery, and high patient satisfaction compared with traditional open microscopic technique.

## Figures and Tables

**Table 1:** Patient demographics and characteristics.

	Endoscopic	Tubular	Microscopic	*p v*alue
Number of patients (N=270)	164	34	72	
Levels (N=315) (1 level/ 2 level/ 3 level)	188 (144/16/4)	40 (28/6/0)	87 (60/9/3)	
Average Age (years)	53.22±3.5	61.80±7.81	59.32±8.28	NS
Gender (male/female)	52/112	10/24	21/51	NS
BMI	28.1±3.4	27.4±3.5	23.2±3.7	NS
Preoperative VAS(back pain)	5.97±2.77	6.61±2.46	5.09±2.84	NS
Preoperative VAS(Leg pain)	7.01± 2.31	7.38±2.40	6.47±2.73	NS
Preoperative ODI	69.8±5.4	68.6±5.8	56..3±6.1	NS
Spinal canal dimension (mm^2^)	81.67±31.30	89.07±40.16	93.52±44.80	NS
Mean follow up duration (months)	6.42±2.68	6.21±3.54	6.32±4.82	NS
Preoperative serum CPK (IU/L)	109.73±46.21	107.2±53.11	99.11±46.44	NS

NS=not significant; BMI= Body Mass Index; VAS=Visual Analogue Scale; ODI=Oswestry Disability Index;

CPK=creatine phosphokinase.

**Table 2:** The Comparison between 3 different decompressive techniques.

	Endoscopic (N=164)	Tubular (N=34)	Microscopic (N=72)
Anesthesia	Epidural (N=52) or General (N=112)	Epidural	Epidural
Skin incision (mm)	10	16-18	25-35
Retractor for working space	Ø 10mm Cannula	Ø 1.6~1.8mm Tube	Taylor retractor
Operative instruments	Ø 3.5, 4.5mm burr Endoscopic Kerrison punch (3, 4, 5 mm)	Ø 5, 6mm burr Kerrison punch (3,5 mm)	
Hemostasis	Radiofrequency, Irrigative pressure	Bipolar, Suction	

**Table 3:** The change of spinal canal dimension (mm^2^).

	Pre Mean(SD)	Post Mean(SD)	*p *value
Endoscopic	81.67±31.30	164.30±53.82	≤0.001*∗*
Tubular	89.07±40.16	153.81±67.9	≤0.001*∗*
Microscopic	93.52±44.80	179.16±52.72	≤0.001*∗*

*∗*=statistically significant.

**Table 4:** Comparison of surgical outcome.

	Endoscopic (N=164)	Tubular (N=34)	Microscopic (N=72)	*p* value
Avg EBL(ml)	35.34±28.87^†^	72±23.21(44-350)	134.3±35.34	0.087
Avg. surgery time (minutes/ level)	84.17±34.70	66.12±15.93	52.22±19.07	≤0.001*∗*
Avg. hospital stay (days)	2.12±1.68	2.83±1.99	4.85±1.86	≤0.001*∗*
Serum CPK (IU/L) ( POD#1 day – Preoperative)	66.38±63.61	137.5±101.00	120 ±116.89	0.030*∗*
Perioperative complication Rate (% of patients)	7.9%	8.8%	8.3%	NS
	Dura tear (4) Dysthesia (7) Motor weakness (1) Disc recur(1)	Postop. Hematoma (1) Dysthsia(1) Disc Recur(1)	Postop. Hematoma(1) Dysthesia(4) Dura tear (1)	

NS=not significant; Avg=average; EBL=estimated blood loss; CPK=creatine phosphokinase; POD=postoperative day; *∗*=statistically significant, †=only hemovac drainage.

## Data Availability

The data used to support the findings of this study are available from the corresponding author upon request.

## References

[1] Verbiest H. (1977). Results of surgical treatment of idiopathic developmental stenosis of the lumbar vertebral canal. A review of twenty-seven years’ experience. *The Journal of Bone & Joint Surgery (British Volume)*.

[2] Hong S.-W., Choi K. Y., Ahn Y. (2011). A comparison of unilateral and bilateral laminotomies for decompression of L4–L5 spinal stenosis. *The Spine Journal*.

[3] Arai Y., Hirai T., Yoshii T. (2014). A prospective comparative study of 2 minimally invasive decompression procedures for lumbar spinal canal stenosis. *The Spine Journal*.

[4] Thomé C., Zevgaridis D., Leheta O. (2005). Outcome after less-invasive decompression of lumbar spinal stenosis: a randomized comparison of unilateral laminotomy, bilateral laminotomy, and laminectomy. *Journal of Neurosurgery: Spine*.

[5] Phan K., Mobbs R. J. (2016). Minimally invasive versus open laminectomy for lumbar stenosis. *The Spine Journal*.

[6] Overdevest G. M., Jacobs W., Vleggeert-Lankamp C., Thomé C., Gunzburg R., Peul W. (2015). Effectiveness of posterior decompression techniques compared with conventional laminectomy for lumbar stenosis. *Cochrane Database of Systematic Reviews*.

[7] Rahman M., Summers L. E., Richter B., Mimran R. I., Jacob R. P. (2008). Comparison of techniques for decompressive lumbar laminectomy: the minimally invasive versus the ‘classic’ open approach. *Minimally Invasive Neurosurgery*.

[8] Oertel M. F., Ryang Y., Korinth M. C., Gilsbach J. M., Rohde V. (2006). Long-term results of microsurgical treatment of lumbar spinal stenosis by unilateral laminotomy for bilateral decompression. *Neurosurgery*.

[9] Kim H. S., Paudel B., Jang J. S. (2017). Percutaneous full endoscopic bilateral lumbar decompression of spinal stenosis through uniportal-contralateral approach: techniques and preliminary results. *World Neurosurgery*.

[10] Hwang J. H., Park W. M., Park C. W. (2017). Contralateral interlaminar keyhole percutaneous endoscopic lumbar surgery in patients with unilateral radiculopathy. *World Neurosurgery*.

[11] Komp M., Hahn P., Oezdemir S. (2015). Bilateral spinal decompression of lumbar central stenosis with the full-endoscopic interlaminar versus microsurgical laminotomy technique: A prospective, randomized, controlled study. *Pain Physician*.

[12] Ruetten S., Komp M., Merk H., Godolias G. (2009). Surgical treatment for lumbar lateral recess stenosis with the full-endoscopic interlaminar approach versus conventional microsurgical technique: a prospective, randomized, controlled study. *Journal of Neurosurgery*.

[13] Lee C., Yoon K., Jun J. (2018). Percutaneous endoscopic laminotomy with flavectomy by uniportal, unilateral approach for the lumbar canal or lateral recess stenosis. *World Neurosurgery*.

[14] Palmer S., Turner R., Palmer R. (2002). Bilateral decompression of lumbar spinal stenosis involving a unilateral approach with microscope and tubular retractor system. *Journal of Neurosurgery*.

[15] Moisi M., Fisahn C., Tkachenko L. (2016). Unilateral laminotomy with bilateral spinal canal decompression for lumbar stenosis: a technical note. *Cureus*.

[16] Arts M., Brand R., van der Kallen B., Lycklama à Nijeholt G., Peul W. (2011). Does minimally invasive lumbar disc surgery result in less muscle injury than conventional surgery? A randomized controlled trial. *European Spine Journal*.

[17] Hatta Y., Shiraishi T., Sakamoto A. (2009). Muscle-preserving interlaminar decompression for the lumbar spine. *The Spine Journal*.

[18] Ho Y.-H., Tu Y.-K., Hsiao C.-K., Chang C.-H. (2015). Outcomes after minimally invasive lumbar decompression: a biomechanical comparison of unilateral and bilateral laminotomies. *BMC Musculoskeletal Disorders*.

[19] Ahn Y., Oh H.-K., Kim H., Lee S.-H., Lee H.-N. (2014). Percutaneous endoscopic lumbar foraminotomy: An advanced surgical technique and clinical outcomes. *Neurosurgery*.

[20] Knight M. T. N., Jago I., Norris C., Midwinter L., Boynes C., Acu L. (2014). Transforaminal endoscopic lumbar decompression & foraminoplasty: a 10 year prospective survivability outcome study of the treatment of foraminal stenosis and failed back surgery. *International Journal of Spine Surgery*.

[21] Choi K.-C., Kim J.-S., Lee D. C., Park C.-K. (2017). Percutaneous endoscopic lumbar discectomy: Minimally invasive technique for multiple episodes of lumbar disc herniation. *BMC Musculoskeletal Disorders*.

[22] Lee C.-W., Yoon K.-J., Ha S.-S., Kang J.-K. (2016). Foraminoplastic superior vertebral notch approach with reamers in percutaneous endoscopic lumbar discectomy: technical note and clinical outcome in limited indications of percutaneous endoscopic lumbar discectomy. *Journal of Korean Neurosurgical Society*.

[23] Kim H. S., Park J. Y. (2013). Comparative assessment of different percutaneous endoscopic interlaminar lumbar discectomy (PEID) techniques. *Pain Physician*.

[24] Benzel E. C., Orr R. D. (2011). A steep learning curve is a good thing!. *The Spine Journal*.

[25] Anichini G., Landi A., Caporlingua F. (2015). Lumbar endoscopic microdiscectomy: where are we now? an updated literature review focused on clinical outcome, complications, and rate of recurrence. *BioMed Research International*.

[26] Cong L., Zhu Y., Tu G. (2016). A meta-analysis of endoscopic discectomy versus open discectomy for symptomatic lumbar disk herniation. *European Spine Journal*.

[27] Wang B., Lü G., Patel A. A., Ren P., Cheng I. (2011). An evaluation of the learning curve for a complex surgical technique: the full endoscopic interlaminar approach for lumbar disc herniations. *The Spine Journal*.

[28] Ahn J., Iqbal A., Manning B. T. (2015). Minimally invasive lumbar decompression--the surgical learning curve. *The Spine Journal*.

[29] He J., Xiao S., Wu Z., Yuan Z. (2016). Microendoscopic discectomy versus open discectomy for lumbar disc herniation: a meta-analysis. *European Spine Journal*.

[30] Sairyo K., Sakai T., Higashino K., Inoue M., Yasui N., Dezawa A. (2010). Complications of endoscopic lumbar decompression surgery. *Minimally Invasive Neurosurgery*.

[31] Belykh E., Giers M. B., Preul M. C., Theodore N., Byvaltsev V. (2016). Prospective comparison of microsurgical, tubular-based endoscopic, and endoscopically assisted diskectomies: clinical effectiveness and complications in railway workers. *World Neurosurgery*.

[32] Sasaoka R., Nakamura H., Konishi S. (2006). Objective assessment of reduced invasiveness in MED. Compared with conventional one-level laminotomy. *European Spine Journal*.

[33] Fu Y., Zeng B., Xu J. (2008). Long-term outcomes of two different decompressive techniques for lumbar spinal stenosis. *The Spine Journal*.

[34] Iwatsuki K., Yoshimine T., Aoki M. (2007). Bilateral interlaminar fenestration and unroofing for the decompression of nerve roots by using a unilateral approach in lumbar canal stenosis. *World Neurosurgery*.

[35] Kim H. S., Patel R., Paudel B. (2017). Early outcomes of endoscopic contralateral foraminal and lateral recess decompression via an interlaminar approach in patients with unilateral radiculopathy from unilateral foraminal stenosis. *World Neurosurgery*.

[36] Ruetten S., Komp M., Merk H., Godolias G. (2007). Use of newly developed instruments and endoscopes: Full-endoscopic resection of lumbar disc herniations via the interlaminar and lateral transforaminal approach. *Journal of Neurosurgery: Spine*.

[37] Hwa Eum J., Hwa Heo D., Son S. K., Park C. K. (2016). Percutaneous biportal endoscopic decompression for lumbar spinal stenosis: a technical note and preliminary clinical results. *Journal of Neurosurgery: Spine*.

[38] Krzok G., Telfeian A. E., Wagner R., Hofstetter C. P., Iprenburg M. (2017). Contralateral facet-sparing sublaminar endoscopic foraminotomy for the treatment of lumbar lateral recess stenosis: technical note. *Journal of Spine Surgery*.

[39] Akçakaya M. O., Yörükoğlu A. G., Aydoseli A. (2016). Serum creatine phosphokinase levels as an indicator of muscle injury following lumbar disc surgery: Comparison of fully endoscopic discectomy and microdiscectomy. *Clinical Neurology and Neurosurgery*.

[40] Pan L., Zhang P., Yin Q. (2014). Comparison of tissue damages caused by endoscopic lumbar discectomy and traditional lumbar discectomy: a randomised controlled trial. *International Journal of Surgery*.

[41] Kim K., Isu T., Sugawara A., Matsumoto R., Isobe M. (2008). Comparison of the effect of 3 different approaches to the lumbar spinal canal on postoperative paraspinal muscle damage. *World Neurosurgery*.

[42] Lee D. Y., Lee S.-H. (2008). Learning curve for percutaneous endoscopic lumbar discectomy. *Neurol. Med. Chir. (Tokyo)*.

[43] Choi D., Choi C., Jung J., Lee S., Kim Y. (2016). Learning curve associated with complications in biportal endoscopic spinal surgery: challenges and strategies. *Asian Spine Journal*.

[44] Podichetty V. K., Spears J., Isaacs R. E., Booher J., Biscup R. S. (2006). Complications associated with minimally invasive decompression for lumbar spinal stenosis. *Journal of Spinal Disorders & Techniques*.

[45] Komp M., Hahn P., Ozdemir S. (2014). Operation of lumbar zygoapophyseal joint cysts using a full-endoscopic interlaminar and transforaminal approach. *Surgical Innovation*.

